# Boosting Probabilistic Graphical Model Inference by Incorporating Prior Knowledge from Multiple Sources

**DOI:** 10.1371/journal.pone.0067410

**Published:** 2013-06-24

**Authors:** Paurush Praveen, Holger Fröhlich

**Affiliations:** University of Bonn, Bonn-Aachen International Center for IT, Bonn, Germany; Semmelweis University, Hungary

## Abstract

Inferring regulatory networks from experimental data via probabilistic graphical models is a popular framework to gain insights into biological systems. However, the inherent noise in experimental data coupled with a limited sample size reduces the performance of network reverse engineering. Prior knowledge from existing sources of biological information can address this low signal to noise problem by biasing the network inference towards biologically plausible network structures. Although integrating various sources of information is desirable, their heterogeneous nature makes this task challenging. We propose two computational methods to incorporate various information sources into a probabilistic consensus structure prior to be used in graphical model inference. Our first model, called Latent Factor Model (LFM), assumes a high degree of correlation among external information sources and reconstructs a hidden variable as a common source in a Bayesian manner. The second model, a Noisy-OR, picks up the strongest support for an interaction among information sources in a probabilistic fashion. Our extensive computational studies on KEGG signaling pathways as well as on gene expression data from breast cancer and yeast heat shock response reveal that both approaches can significantly enhance the reconstruction accuracy of Bayesian Networks compared to other competing methods as well as to the situation without any prior. Our framework allows for using diverse information sources, like pathway databases, GO terms and protein domain data, etc. and is flexible enough to integrate new sources, if available.

## Introduction

Probabilistic graphical models, like (Dynamic) Bayesian Networks and Gaussian Graphical Models, have turned out to be useful for extracting meaningful biological insights from experimental data in life science research. These models can infer features of cellular networks in a data driven manner [1]–[4]. However, network inference from experimental data is challenging because of the typical low signal to noise ratio [5]. High throughput data like microarray is very high dimensional coupled with a typical low number of replicates and noisy measurements. Reverse engineering of regulatory network on the basis of such data is hence challenging and often fails to reach the desired level of accuracy. To deal with this problem one can either work at experimental level by increasing the sample size, which is practically difficult, or at the inference level by embedding biological background knowledge.

Integrating known information from databases and biological literature as prior knowledge thus appears to be beneficial. However, biological knowledge covers many different aspects and is widely distributed across multiple knowledge resources, such as pathway databases [6]–[8], Gene Ontology [9] and others. Hence, integrating this heterogenous information into the learning process is not straight forward.

In the past most authors have concentrated on integrating *one* particular information resource into the learning process [10]–[15]: E.g. gene regulatory networks were inferred from a combination of gene expression data with transcription factor binding motifs in promoter sequences [11], protein-protein interactions [12], evolutionary information [13], KEGG pathways [14] and GO anotation [15].

On the technical side several approaches for integrating prior knowledge into the inference of probabilistic graphical models have been published: In [16] and [17] the authors only generate candidate structures with significance above a certain threshold according to prior knowledge. Another idea is to introduce a probabilistic Bayesian prior over network structures. E.g. Fröhlich *et al.* [18] introduced a prior for individual edges based on an a-priori assumed degree of belief. Mukherjee *et al.* [19] describes a more general set of priors, which can also capture global network properties, such as scale-free behavior. Wehrli and Husmeier [20] use a similar form of prior as Fröhlich *et al.*, but additionally combine multiple information sources via a linear weighting scheme. The weights are sampled together with the rest of the parameters and the network structure in a specifically designed Markov Chain Monte Carlo algorithm for Bayesian Network inference. In contrast, Gao and Wang [21] treat different information sources as statistically independent, and consequently the overall prior is just the product over the priors for the individual information sources. The advantage of the approach is that it is independent from a particular class of probabilistic network models (e.g. Bayesian Networks). The limitation is its strong assumption of non-conditional statistical independence of information sources, which in reality is unlikely, since biological knowledge in different databases is not orthogonal to each other.

The focus of this paper is on construction of consensus priors from multiple, heterogenous knowledge sources. These consensus priors can then be incorporated for learning probabilistic graphical models (e.g. Bayesian Networks) from experimental data. We are at this point aware of the fact that there is a broad literature on (probabilistic) data integration [22], [23], which goes beyond our specific question and covers a large variety of different aspects [24]–[27].

In this paper we propose two alternative ways to integrate heterogenous information from multiple knowledge sources into a consensus prior. Our first model, which we call Latent Factor Model (LFM), relies on the idea of a generative process for the individual information sources and uses Bayesian inference to estimate a consensus prior. The second model integrates different information sources via a Noisy-OR gate. Both models are very general and do neither rely on a specific probabilistic model nor on a specific inference procedure. We exemplify the benefit of our consensus priors for the inference of Bayesian Networks.

## Materials and Methods

### 1.1 Edge-wise Priors for Data Driven Network Inference

Let 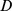 denote our experimental data and 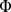 the network graph (represented by an 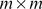 adjacency matrix), which we would like to infer from this data. According to Bayes' rule the probability of network 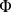 given data 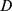 is given as
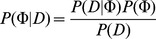
(1)where 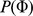 is the prior. We assume that 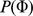 can be decomposed into



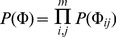
(2)e.g.
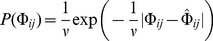
(3)where 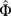 is a matrix of prior edge confidences [18]. A value of 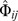 close to 1 indicates a high prior degree of belief in the existence of the edge 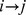. Our purpose is to compile 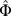 in a consistent manner from 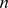 available information sources. We suppose that each of these sources allows for obtaining an edge confidence matrix by itself, i.e. altogether with 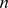 information sources we have 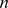 edge confidence matrices 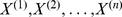.

### 1.2 Latent Factor Model (LFM)

**Figure 1:**
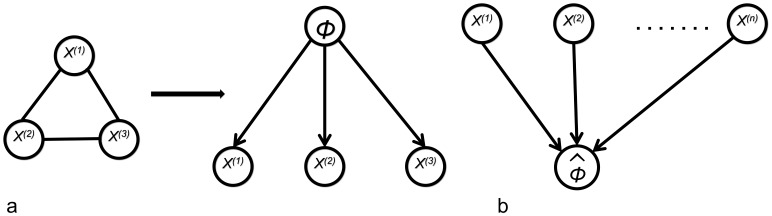
Graphical models representing approaches. (a) A general Latent Factor Model (LFM). The random variables 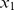, 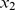 and 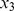 are highly related variables (left) and an assumption that these related random variables originate from a common, true but unknown variable 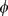 results a bayesian network (right) in case of networks 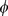 is the true but unknown network. **(b)** A generalized view of a Noisy-OR model showing the relation between causes 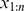 and effect 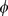 through a Noisy-OR function.

The Latent Factor Model is based on the idea that the prior information encoded in matrices 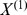, 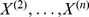 all originate from the true but unknown network 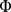 (Figure 1a). This specifically implies that direct correlations between edge confidences across matrices can be explained by this hidden dependency. In other words 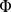 is a latent factor explaining correlations between the 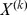 (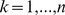). We use this notion to conduct joint Bayesian inference on 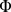 as well as additional parameters 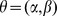 given 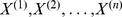:
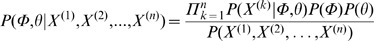
(4)


The idea behind this equation is that we can identify 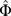 with the posterior 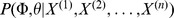. In other words the prior edge confidences 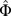 are identical to the posterior edge probabilities learned from our 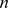 information sources 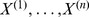.

The entries of each matrix 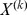 can be assumed to follow beta distributions. More specifically we have:
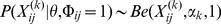
(5)

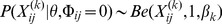
(6)


and 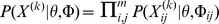


Please note that 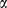 and 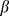 are vectors and 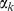 and 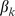 are the specific values for source 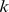. If the values in matrix 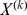 all either very high (close to 1) or low (close to 0) parameters 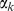 and 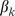 will have a large magnitude. Consequently, 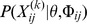 will be large, i.e. source 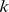 has a large impact. On the other hand, if values in 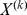 are rather uniformly distributed, parameters 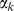 and 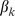 will be close to 1, which implies 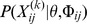 to be close to 0. Thus such an information source has only small impact. By introducing source specific beta distribution parameters we are hence able to weight these source individually.

We employ an adaptive Markov Chain Monte Carlo (MCMC) strategy [28] to learn the latent variable 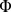 together with parameters 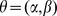. For this purpose we define MCMC moves in network space as well as in parameter space. More specifically, in network space MCMC moves are edge insertion, deletion and reversal. In parameter space 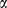 and 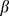 are adapted on log-scale using a multivariate Gaussian transition kernel. This is done every 10th iteration. The covariance matrix of the Gaussian transition kernel is initialized to the identity matrix and every 100th iteration updated to the empirical covariance matrix. The number of burn-in steps used is 100000 and number of sampling iterations is 500000 for our MCMC algorithm here (see example convergence plot in Figure S2 in File S1).

### 1.3 Noisy-OR Model (NOM)

The Noisy-OR represents a non-deterministic disjunctive relation between an effect and its possible causes and has been extensively used in artificial intelligence [29]. The Noisy-OR model assumes that the relation among the causes and the effect is not-deterministic, allowing the presence of the effect in absence of any of the modeled causes. The Noisy-OR principle is governed by two hallmarks: First, each cause has a probability to produce the effect and second, the probability of each cause being sufficient to produce the effect is independent of the presence of other causes (Figure 1b).

In our case 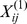, 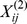, …, 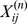 are interpreted as causes and 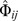 as effect. The link between both is given by
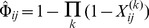
(7)


In consequence 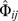 becomes close to 1, if the edge 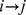 has a high confidence in at least one information source, because then the product gets close to 0. Hence, in the Noisy-OR model high edge confidences in one information source can overrule low confidences in other information sources. This is in contrast to the LFM model, where a high level of agreement between information sources is required in order to achieve high values in 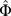.

In addition to the above described Noisy-OR model, which integrates edge confidences directly into the consensus prior, we also experimented with a variant based on relative ranks, which is in the spirit of Marbach *et al.* [26]: Within each matrix 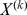 we first assigned each edge confidence 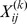 to its rank 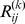 in descending order. Then we converted these absolute ranks into relative ranks by dividing each rank value by the maximum rank:
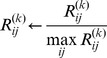
(8)


Matrices 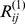, 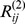, …, 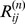 consisting of relative ranks were then considered in Eq. (7) rather than the original matrices 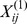, 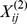, …, 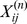. We call this method NOM.RNK in the following.

### 1.4 Information Sources

In this work we employed GO annotation, two pathways databases (KEGG, PathwayCommons), protein domain annotation (InterPro – [30]) and protein domain interactions (DOMINE – [31]) as sources of prior information. According to each of these information sources we calculated for a pair of proteins 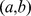 a [0, 1] normalized similarity, which we interpreted as edge confidence. Briefly, for GO annotation we used the default similarity measure for gene products implemented in the R-package GOSim [32], which resembles the functional similarity proposed by Schlicket *et al.* [33] on the basis of the information theoretic GO term proximity measure by Lin [34]. Protein domain annotation was compared on the basis of a binary vector representation via the cosine similarity. The relative frequency of interacting protein domain pairs was taken as another confidence measure for an edge 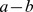. Finally, network information was integrated by computing shortest path distances between pairs of proteins. Details about our similarity measures and their calculation can be found in the supplemental material (Supplement text and Figure S1 in File S1).

## Results

### 2.1 Correlation of Prior Edge Confidences with True Biological Network

**Network Sampling:** In a first series of validation experiments we looked, in how far the true network could be recovered purely from the inferred prior edge confidence matrix 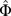 after a applying a certain threshold. For this purpose we generated 10 networks with 10, 20, 40 and 60 nodes each. These networks later on served as our ground truth. To obtain our ground truth networks we parsed XML files of all KEGG signaling pathways and converted them into graphs via the R-package KEGGgraph [35]. Then we randomly picked one of these graphs and performed a random walk starting from a randomly selected core node. The random walk was stopped once a predefined number of distinct nodes had been visited, and the corresponding sub-network was returned as a ground truth network (Figure S9 in File S1).

To evaluate the performance of a prior relative to the ground truth network we looked at sensitivity and specificity at different cutoffs for edge confidences. In addition we also computed the balanced accuracy ( = average of sensitivity and specificity) at each cutoff. We then defined the *optimal balanced accuracy* (oBAC) to be the maximum balanced accuracy over all these cutoffs.

**Simulated Information Sources:** In order to better understand the principal behavior of our LFM, NOM and NOM.RNK methods we first simulated matrices 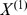, 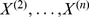 by sampling from 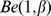 and 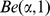 distributions according Eq. 6. The whole simulation was repeated 10 times for different parameter combinations and network sizes 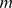. We compared our LFM, NOM and NOM.RNK approaches against a set of other proposed priors namely.

1. an independent prior (IP), which just takes the product of all matrices 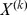 (mimicking the method by Gao et al. [21])
2. a variant of IP working on relative ranks (IP.RNK) in the same way as described for the NOM method
3. an unweighted average prior (MP), which takes the arithmetic mean of all matrices 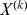

4. a variant of MP, which works on relative ranks (MP.RNK) and is thus identical with the approach proposed by Marbach et al. [26]

To understand the dependency on 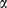 and 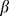 we first varied both parameters in the range 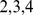 and fixed 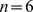 for networks with 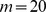 nodes. Our results (Figures S3, S4) indicate a dependency of the priors, on the beta distribution shape parameters. Under most parameter settings the methods using relative ranks performed better than their counterparts using raw edge confidences. This was not true for NOM versus NOM.RNK, however, were the opposite behavior was observed: NOM.RNK compared to NOM lacks specificity. Almost all the models performed better for highly correlated sources (i.e. higher 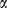 and 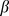 values – see Figure S5 in File S1). However, the LFM model performed well even with an overall low correlation among sources, which can be interpreted by the ability of the approach to down-weight uninformative/weakly correlated sources. The same held true for MP.RNK. IP was comparable to the other methods for only two parameter combinations 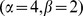 and 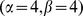. In both cases numerically the beta distribution yields relatively high values in the 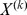 matrices, hence the product does not as quickly tend to 0 as with lower values. NOM could beat LFM only for 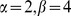. In this case confidence values for non-existing edges are relatively concentrated around 0, and LFM lacks sensitivity. On the other hand LFM performed significantly better than NOM for 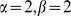 and 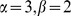 and 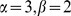. In these cases confidence values for existing edges are relatively high, and NOM lacks specificity. In general it was observable that LFM, MP, MP.RNK, IP and IP.RNK are extremely specific methods, whereas NOM is highly sensitive. Consequently LFM gives the best results in terms of balanced accuracy at low edge probability cut-offs whereas, the NOM does the same at higher cut-offs. The correlation of entries in matrices 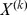 were dependent on the beta distribution parameters (Figure S5 in File S1). For example 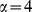, 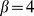 yielded high correlations (median 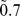),whereas 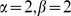 lead to much weaker ones (median 0.2).

We also simulated the network reconstruction performance for different number 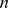 of sources for networks with 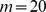 nodes and 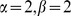. In this situation we could observe that increasing the number of sources helped to improve the accuracy for most methods (Figure S6 in File S1). The oBAC of our methods were similar to those of the other approaches for a low number of sources (1, 2 and 3 sources). However, with an increasing number of sources (4, 5 and 6) the performance of LFM increased constantly. For NOM an optimum was reached for 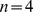 sources, after which the performance declined again, suggesting an increasing loss of specificity.

Decreasing the number of network nodes from 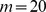 to 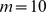 yielded a drastic performance loss of LFM (Figure S7 in File S1). This may be explained be the fact that the LFM method learns from the entries in the matrices 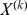. The larger these matrices, the more independent observations LFM has to learn from. In contrast, increasing the number of network nodes from 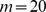 to 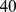 and 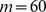 for 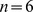 sources and 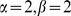 did not influence the previously observed good performance of LFM significantly.

**Figure 2:**
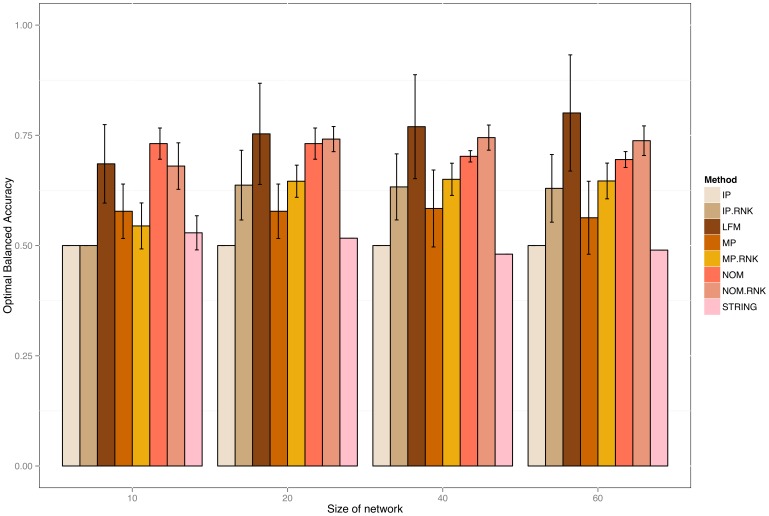
Plot showing the balanced accuracies of networks with varying number of nodes (20, 40 and 60) created just from different kinds of prior knowledge. The networks were extracted from KEGG via random walks. The plot shows the effect of size of network of different priors and also compares them to the knowledge from STRING database.

**Weighting of Information Sources:** We tested, in how far the automatic weighting of sources provided by the LFM method was able to filter out irrelevant/noisy information. For this purpose we added an additional artificial source, which contained values sampled uniform randomly between 0 and 1. Figure 2 depicts the posterior expectations for 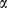 and 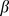 parameters, which were retrieved for individual information sources for 10 sampled networks with 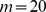 nodes. The picture clearly reveals that the posterior expectation of parameters for the noise source was always close to 1, which indicates an influence close to 0 in the likelihood function (Eq. 6). Hence, the noise source was filtered out effectively.

**Real Information Sources:** In a second round of experiments we constructed prior information for our 10 sampled networks from existing biological knowledge encoded in GO, PathwayCommons, KEGG, InterPro and DOMINE (see section “Information Sources” and Supplements). We ran the whole simulation for networks of different sizes (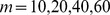).

**Figure 3:**
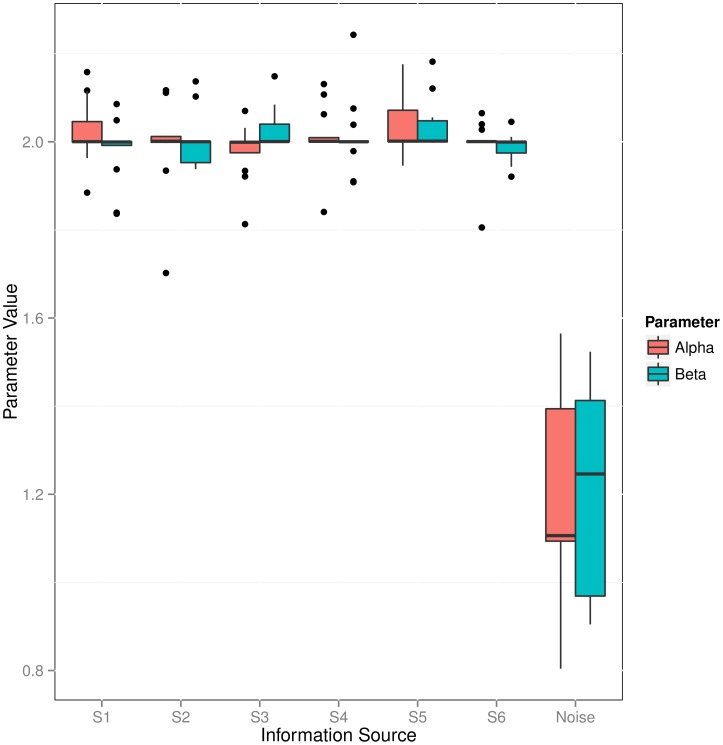
Boxplot of posterior expectation parameters learned for individual information sources in 10 randomly sampled sub-graphs of KEGG pathways of size *m* = 20.

Our studies revealed a significant improvement of our suggested methods (LFM, NOM, NOM.RNK) compared to the other models in all cases (Figure 3 together with Figure S8 and Table S4 in File S1). These findings were underlined by a pairwise Wilcox signed rank test to assess the statistical significance of the observed differences (Table 1). At the same time no statistically significant differences between NOM, LFM and NOM.RNK could be observed in terms of oBAC here. The IP prior revealed a oBAC which was almost constantly at 0.5. The reason for this behavior is that multiplicative nature of the IP method often yields numerically very small values, hence making IP close to a pure sparsity prior.

**Table 1:** Pairwise Wilcoxon test for model performance comparison (false discovery rates) for 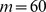.

Methods	IP	IP.RNK	LFM	MP	MP.RNK	NOM	NOM.RNK
IP.RNK	0.0091	–	–	–	–	–	–
LFM	0.0036	0.0036	–	–	–	–	–
MP	0.2503	0.0249	0.0036	–	–	–	–
MP.RNK	0.0036	0.6953	0.0036	0.0036	–	–	–
NOM	0.0036	0.0433	0.0137	0.0036	0.0091	–	–
NOM.RNK	0.0036	0.0333	0.0182	0.0036	0.0137	0.3889	–
STRING	0.0036	0.0068	0.0036	0.1466	0.0036	0.0036	0.0036

For 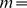 10, 20 and 40 see tables S1, S2, and S3 in file S1.).

We also compared the reconstruction performance of our priors to a reconstruction with confidence scores from the STRING database [36]. The comparison showed a clear and significant advantage of our priors over the STRING in terms of higher oBAC (Figure 3, Table 1 and Tables S1–S3).

Most methods showed a very low dependency on the network size, except for the LFM method, which tended to improve the more nodes the network had. The reason for this behavior could be that the LFM method essentially learns from the entries of the edge confidence matrices. Having larger matrices implies more independent observations to learn from, hence the performance increases.

### 2.2 Enhancement of Data Driven Network Reconstruction Accuracy

**Simulated Data:** We next investigated, in how far our priors could enhance the reconstruction performance of Bayesian Networks learned from data. This serves as an example for the ability to enhance probabilistic graphical model inference using our informative priors. For the purpose of this simulation we used the R-package *catnet*. The *catnet* package implements a dynamic programming approach to exhaustively search through the space of possible network structures and returns a set of best fitting models. The maximum number of parents per network node can be limited to a user specified number (here: 5). From the set of best fitting network structures the one with minimum BIC value was selected. *catnet* allows to specify a Bernoulli distribution prior over network structures:
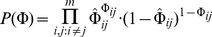
(9)


Please note that the prior is specified in terms of arbitrarily chosen edge-wise probabilities. In any case, network structures learned by *catnet* are directed acyclic graphs.

**Figure 4:**
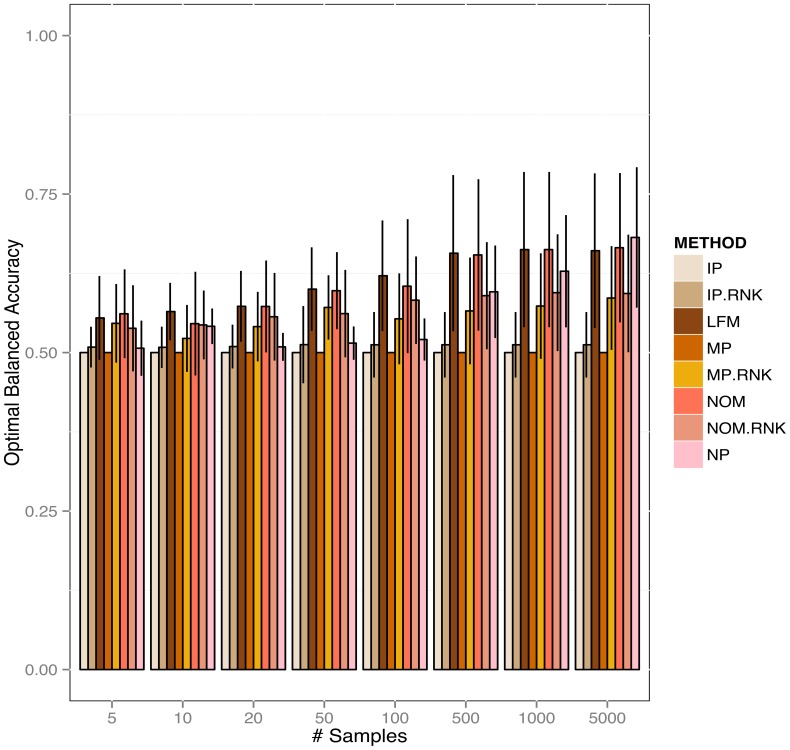
Optimally balanced accuracy for reconstructing networks from simulated categorical data with different kinds of prior (# nodes = 10).

In order to conduct our simulation we sampled 10 graphs (Figure S10 in File S1) with 10 nodes from KEGG signaling pathways (see description in section “Network Sampling”). While doing so, we specifically ensured that only directed acyclic graph structures were generated (others were discarded). For each generated network multinomially distributed data with 3 categories were sampled using the appropriate functions in R-package *catnet*. This was repeated 10 times with different numbers of data points (5, 10, 20, 50, 100, 500, 1000 and 5000 data points per variable). We then tested Bayesian Network inference using the LFM, NOM, IP, MP, IP.RNK, MP.RNK, NOM.RNK priors as well as without any prior (no prior – NP). Performance evaluation of learned network structures was done in terms of sensitivity (true positive rate), specificity (1 - false positive rate) and balanced accuracy (average of sensitivity and specificity). paragraph The results showed a clear positive effect of our priors for biologically relevant sample sizes (Figure 4, rest in S11). Specifically for sample sizes between 20 and 100 LFM, NOM and NOM.RNK were superior to all other methods (FDR 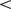 5% for comparison against IP, IP.RNK, MP, NP for all sample sizes) (See Table S5 and S6). The MP.RNK method was the best competing method, but was significantly outperformed by LFM for all sample sizes 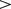 20. The independence prior (IP) in all cases yielded numerical problems, because for 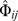 values close to 0 the prior in Eq. 9 on log scale tends to minus infinity. Therefore, IP in all cases produced the same constant results. As expected, for larger sample sizes (1000, 5000 data points) the effect of using an informative prior compared to using no prior at all vanished.

Overall, our proposed methods allowed for a significant improvement in the network reconstruction process compared to using no prior and compared to using the IP, IP.RNK, MP and MP.RNK priors.

**Application to Breast Cancer:** We applied our tested approaches to build informative priors for a sub-sample of the well known breast cancer microarray data set by van't Veer *et al.* [37] contained in *catnet*. The data consists of 1214 genes for 98 patient samples: 34 patients developed distant metastases within 5 years, 44 patients remained disease-free after a period of at least 5 years, 18 patients had BRCA1 germline mutations, and 2 were BRCA2 carriers. We selected 173 differentially expressed genes (FDR cutoff 5%) from this dataset via SAM analysis [38]. From this set of genes we further selected a cluster consisting of 37 genes for network inference via complete linkage clustering.

**Figure 5:**
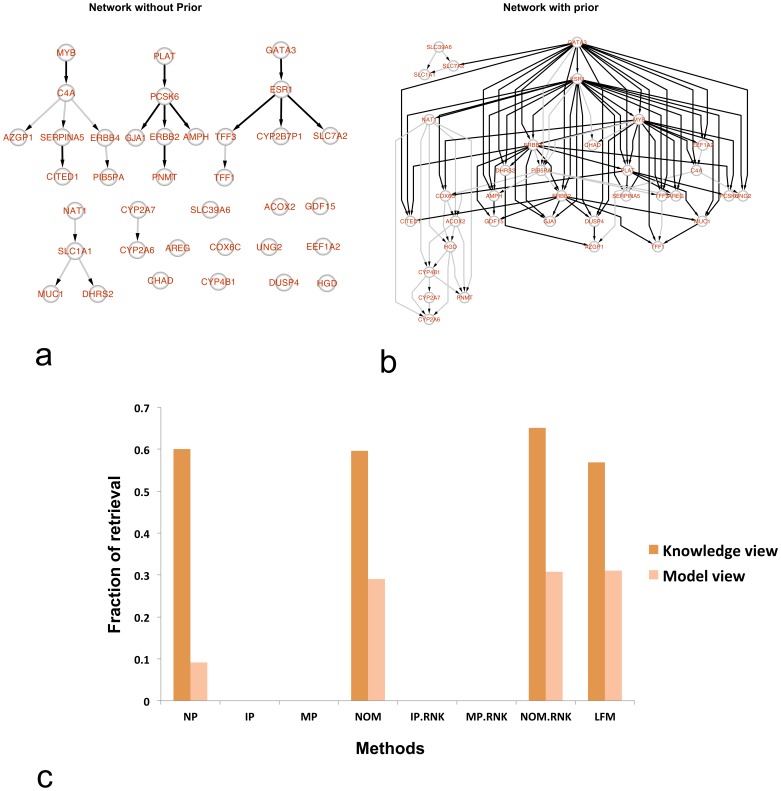
Network reconstruction for the breast cancer data (van't Veer et.al.). (**a**) The reconstructed network from data without using any prior. (**b**) Reconstructed network using the NOM prior. Black edges in the network could be verified with established literature knowledge, whereas the grey edges could not be verified. (**c**) The plot shows the edge recovery of the network from two points of view points: knowledge view = literature network mapped onto reconstructed network; model view = reconstructed edges mapped onto literature network.

Bayesian Network inference via *catnet* was run with restricting the maximal number of parents per network node to 5. This was done once without using any prior and then with the LFM, NOM, NOM.RNK, IP, IP.RNK, MP and MP.RNK priors (Figure 5a and 5b). To compare, we also retrieved a network for these 37 genes purely from literature known interactions via the commercial software MetaCore. The literature network consisted of all shortest paths between the 37 genes, which can be computed purely via literature known interactions (Figure S12 in File S1). That means the literature network mainly consists of indirect interactions.

We asked (i) in how far inferred edges between two nodes could be explained by shortest paths in the literature network (so-called *model view*) and (ii) in how far shortest paths between the 37 genes in the literature network corresponded to paths in the inferred network (so-called *knowledge view*). These two performance measures capture the situation that a) the literature network consists of indirect interactions only and b) there could exist edges in the data, which are so far unknown in the literature for human.

The results showed that Bayesian Network reconstructions using LFM and NOM priors were significantly closer to the established biological knowledge than without using any prior (Figure 5c). On the other hand usage of the other priors did not yield any significant overlap with the literature. With the NOM and NOM.RNK priors more than 60% of the inferred edges could be explained by the literature and around 30% of the literature known paths corresponded to pathways in the inferred network. The fact that the latter percentage is much lower than the fraction of literature explainable edges in the inferred network has several reasons: First, a Bayesian Network can only infer a directed acyclic graph, but literature based networks are typically highly cyclic. Second, Bayesian Networks try to uncover conditional independence relationships in the data. However, not all existing molecular interactions might manifest in such relationships on gene expression level. Third, not all literature reported interactions are guaranteed to exist in the specific cells under investigation.

**Figure 6:**
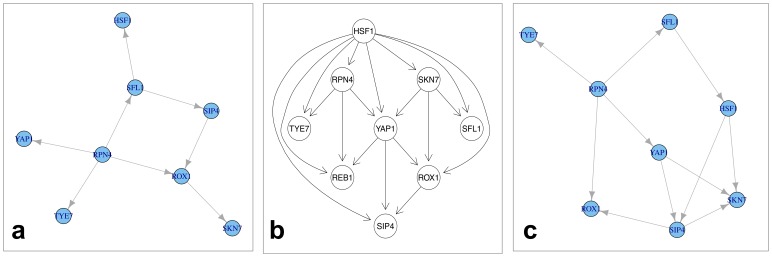
Yeast (*Saccharomyces cerevisiae*) heat-shock response network obtained via Bayesian network reconstruction. (a) Network without any prior knowledge, (b) The gold standard network from YEASTRACT database (c) Network reconstructed with prior knowledge (here: NOM).

**Application to Yeast Heat-Shock Network:** In second application we used our method to infer a network of nine transcription factors (TFs) related to yeast heat-shock response. We used microarray data from GEO (GSE3316), which contains 12 samples. We considered two different sources of established knowledge to compute a consensus prior, namely Gene Ontology (GO) and protein-protein interactions for Yeast obtained from PathwayCommons [7]. Bayesian Network inference was done in a similar manner as described above. After network reconstruction we compared the resulting network against the gold standard network from the YEASTRACT database [39] (Figure 6).

**Figure 7:**
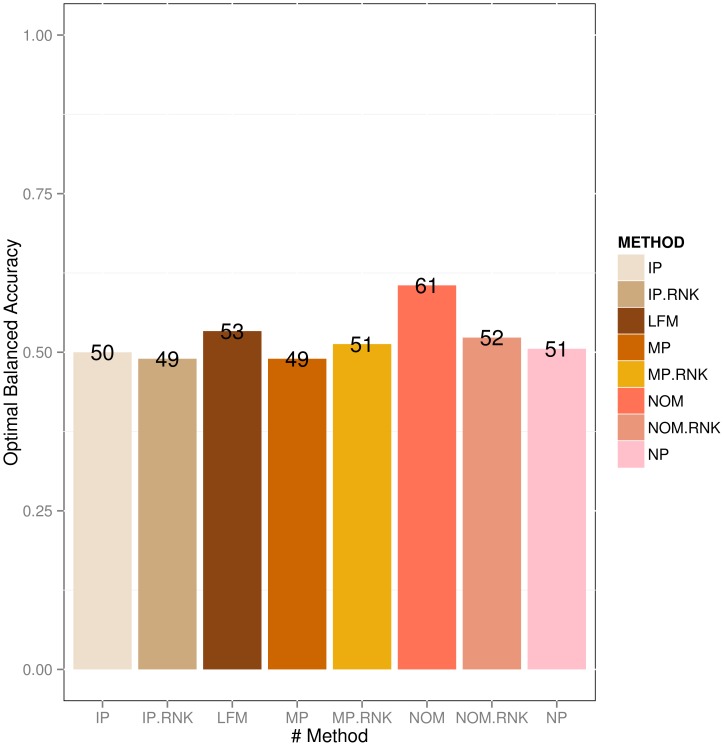
Reconstruction performance of Yeast (*Saccharomyces cerevisiae*) heat-shock response network with Bayesian Networks and different priors (NP = No Prior).

Knowledge integration via the NOM prior lead to an improvement of 10% in terms of balanced accuracy compared to using no prior (Figure 7). The other prior methods (including LFM) did not yield any significant increase in reconstruction performance. The reason for the bad performance of LFM is probably the low number of available knowledge sources combined with a relatively small network size.

## Discussion

We proposed two methods to integrate different, heterogenous sources of biological information in form of a consistent structure prior for probabilistic network inference. Our approach takes into consideration diverse information sources, such as e.g. GO, pathway and protein domain data. Our latent factor model (LFM) is based on the assumption of relatedness of biological information across these data sources. In contrast the Noisy-OR model (NOM) picks up the the strongest support for an interaction from any of the knowledge sources.

Our computational experiments revealed that both of our models yielded priors which were significantly closer to the true biological network than competing methods. Moreover, they could significantly enhance the reconstruction performance of Bayesian Networks compared to a situation without any prior, an independent as well as a mean prior approach. This was true, even if relative ranks were employed, which generally appeared to be beneficial for IP and MP, but not necessarily for NOM. Our methods were also superior to purely using STRING edge confidence scores as prior information. Furthermore, we found that LFM particularly worked particular well, if networks were not too small (Figure 2). Therefore, in case of very small networks and/or sparse prior knowledge NOM appears to be a more robust choice. Moreover, NOM is clearly the computationally cheaper approach and thus should be favored for very large (e.g. genome-scale) networks. Taken together LFM thus appears to be a recommendable choice mainly for medium sized networks, if a sufficient degree of correlation between information sources can be observed.

The current framework allows to include a number of heterogenous information sources and is flexible enough to include new ones. As databases for biological information and annotation grow, a larger amount of correlated information can be compiled into prior knowledge, which ultimately can be utilized to more realistic probabilistic model inference from experimental data.

## Supporting Information

File S1 **Additional plots and tables for the studies on prior knowledge integration.**
(PDF)
