# Supplementary material for: Boosting Probabilistic Graphical Model Inference by Incorporating Prior Knowledge from Multiple Sources
Source: PLoS One. 2013 Jun 24;8(6):e67410. doi: 10.1371/journal.pone.0067410 (PMC3691143; doi:10.1371/journal.pone.0067410)
Supplement: File S1 — Additional plots and tables for the studies on prior knowledge integration. (PDF) [file pone.0067410.s001.pdf]

Boosting Probabilistic Graphical Model Inference by Incorporating Prior Knowledge from Multiple Sources (Supplement)

Paurush Praveen, Holger Fröhlich

May 2, 2013

1 Knowledge sources

The approaches presented here primary consider the following sources of biological knowledge, however new sources can be added if available.

Protein-Protein Interactions

Protein-protein interaction (PPI) data can be instrumental to understand biology at a system-wide level. They present the current knowledge about pairs of proteins that interact in living system and hence can be an important source of information for our approach. PPIs have traditionally been measured using a variety of assays, such as immunoprecipitation and yeast two-hybrid (Y2-H). Such knowledge resides in various databases, like IntAct, HPRD *etc.* The entire set of known interaction referred as interactome can be structured as a graph. We here use interaction data from the PathwaysCommons database [1]. To compute a confidence value for each interaction between a pair of genes/proteins we can work at the level of this graph in different ways: One way is to look at the shortest path distance between the two entities. Another way is to employ diffusion kernels [6]. To calculate the shortest path distance between two nodes the function *sp.between* function based on Dijkstra's algorithm is used from R-package RBGL. The edge confidence is then computed as the inverse shortest path distance.

The diffusion kernel was computed using the R-package pathClass [5] for the entire PathwayCommons graph. However, we observed that the use of the diffusion kernel in place of the shortest path measure did not affect the results much (Figure 1). For all the results in the main document we thus used the inverse shortest path distance as a measure of confidence.

Gene Ontology

The Gene Ontology (GO) has been developed to offer controlled vocabularies for aiding the annotation of molecular attributes for different organisms. Predicting the map of potential physical interactions between proteins by fully exploring the knowledge buried in GO annotations seems a promising approach, because interacting proteins often function in the same biological process. This implies that two proteins acting in the same biological process are more likely to interact than two proteins involved in different processes. Here we use this information based on GO *BiologicalProcess* (BP) annotations. Comparison of individual GO terms was performed via Lin's similarity measure [7]. Based on this gene products were compared via the default method in GOSim [3], which resembles the similarity measure by Schlicker et.al.[10].

Protein Domain Annotation

Hahne et.al [4] and Fröhlich et.al. [2] found that proteins in distinct KEGG pathways are enriched for certain protein domains, i.e. proteins with similar domains are more likely to act in similar biological pathways. The confidence of interaction between two proteins can thus be seen as a function of the similarity of the domain annotations of proteins. Protein domain annotation can be retrieved from the Inter-Pro database [8]. For each protein we constructed a binary vector, where each component represents one Inter-Pro domain. A "1" in a component thus indicates that the protein is annotated with the corresponding domain. Otherwise a "0" is filled in. The similarity between two binary vectors u, v (domain signatures) is presented in terms of the cosine similarity

$$S_{domain} = \frac{\langle u, v \rangle}{\|u\|\|v\|} \tag{1}$$

Domain-Domain Interactions

Two proteins are more likely to interact if they contain domains, which can potentially interact. The DOMINE database collates known and predicted domain–domain interactions [9]. Calculation for edge confidence (I_{AB}) based on the DOMINE database is done as

$$I_{AB} = \frac{H}{D_A.D_B} \tag{2}$$

where H is the number of hit pairs found in the DOMINE database and D_A and D_B are the the number of domains in proteins A and B, respectively.

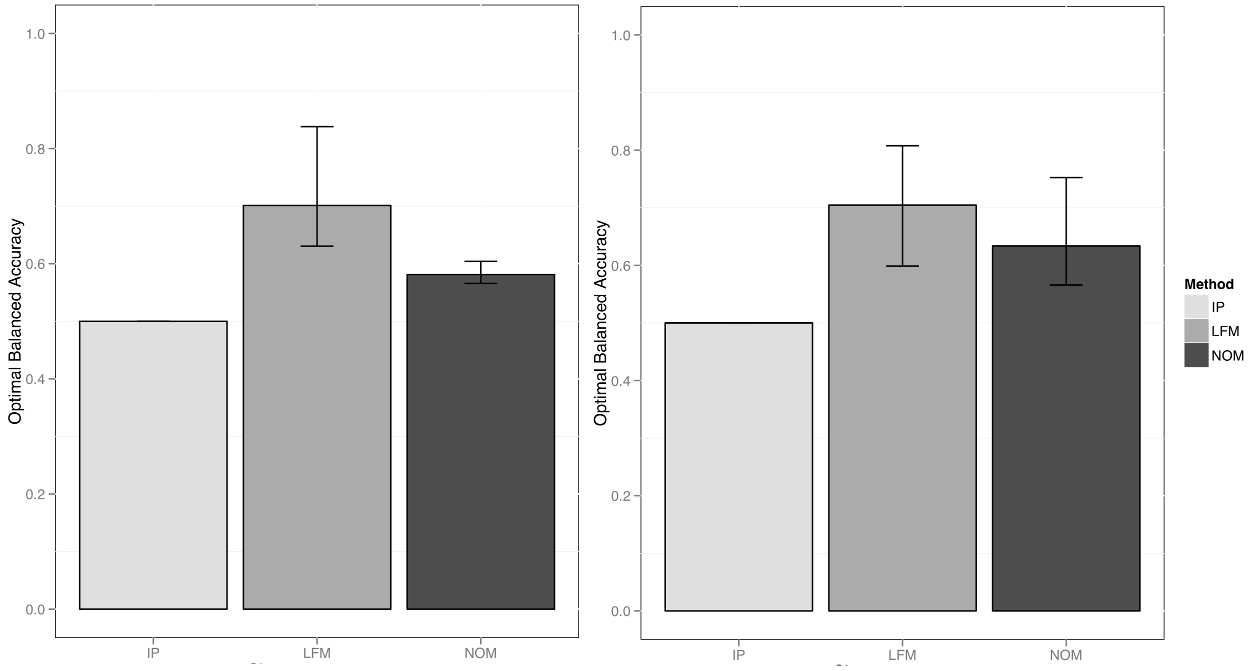

Figure 1: Optimal balanced accuracies achieved for reconstructing KEGG subgraphs (m=20) using graph diffusion kernel (left) and inverse of pairwise shortest path-lengths (right)

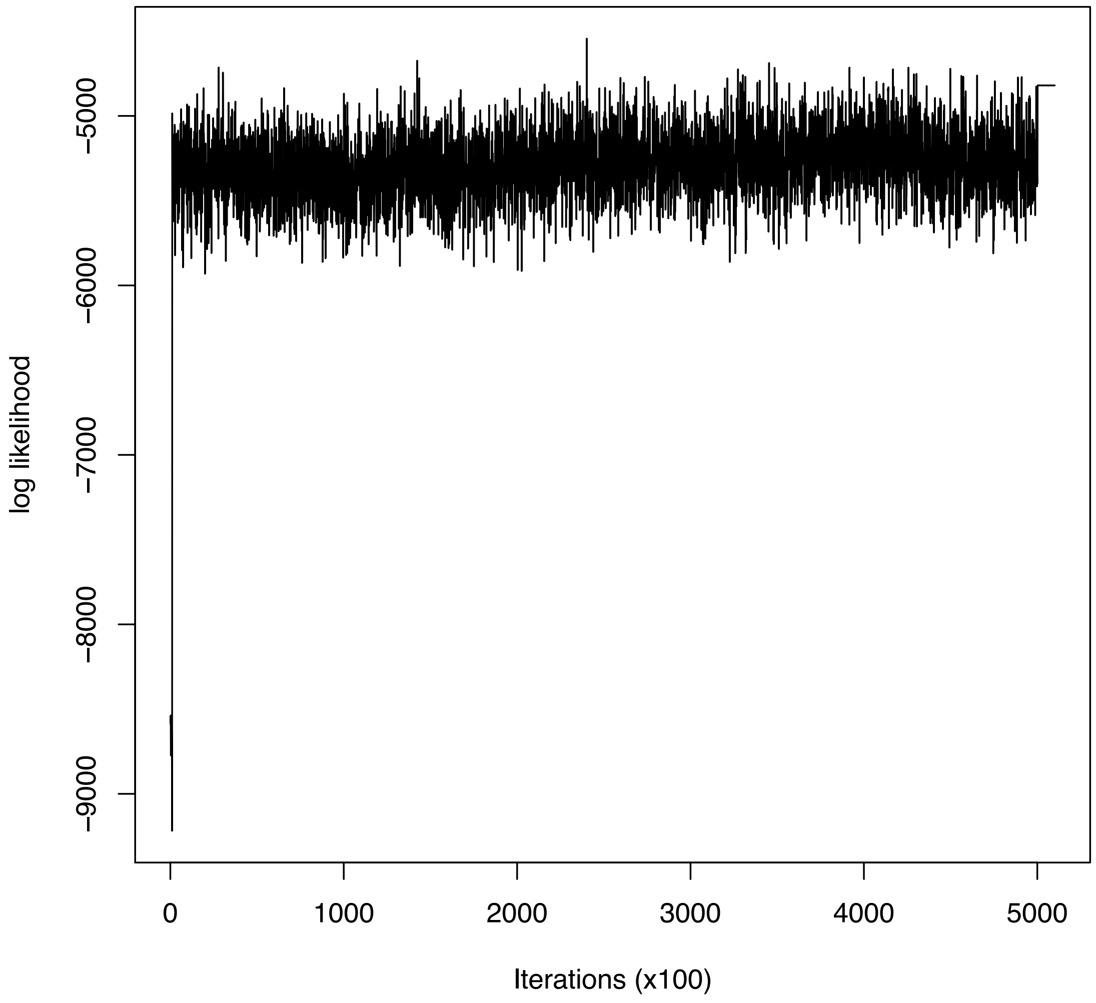

Figure 2: Plot showing the convergence of the MCMC sampler in terms of log likelihoods.

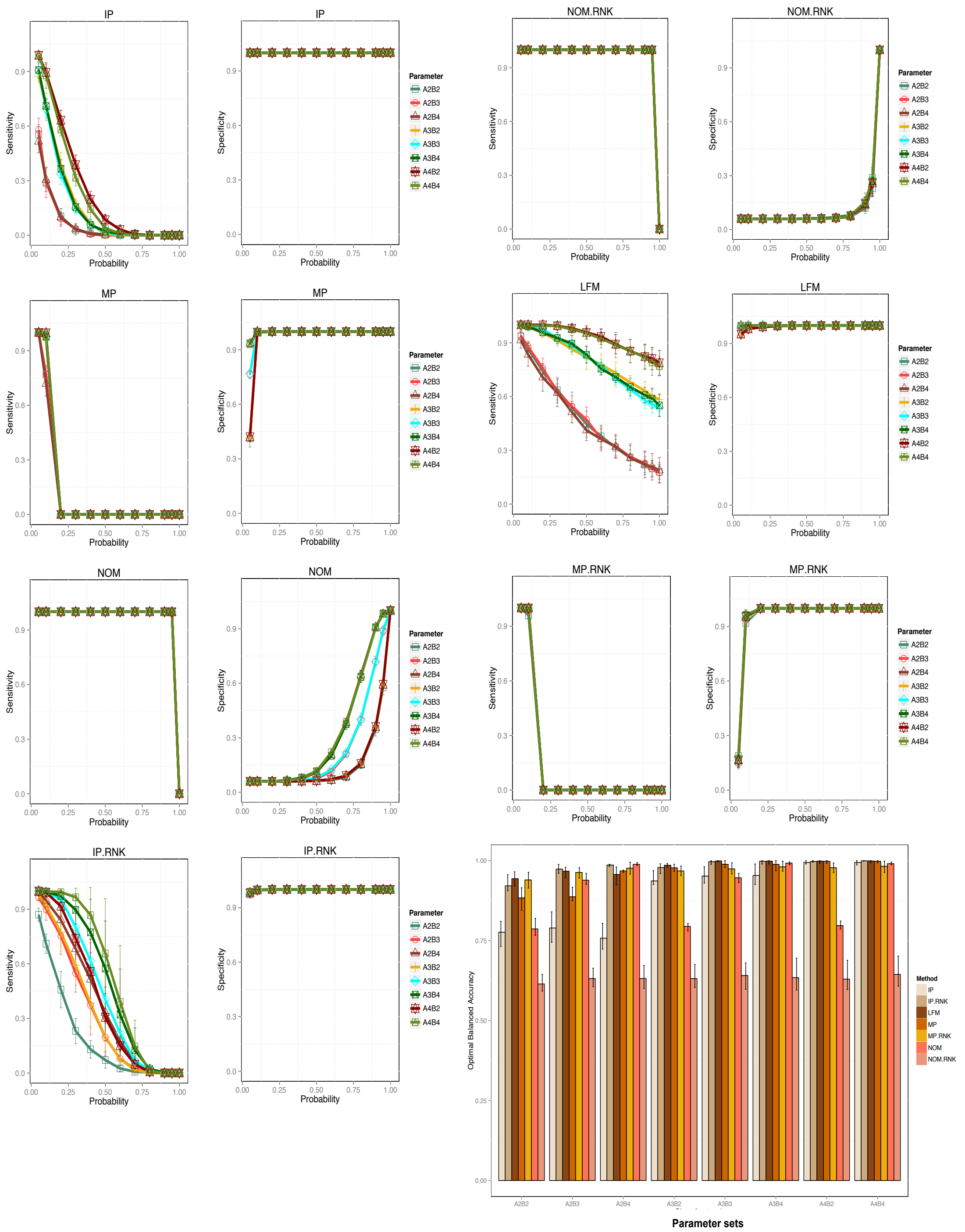

Figure 3: Sensitivity and specificity plots for different set of α and β parameters for artificially generated information source data. The plot shows the behavior of different kinds of priors (top) and the corresponding optimal balanced accuracies (bottom). The number of nodes for all the networks here were 20 (m=20), and 6 information sources were used.

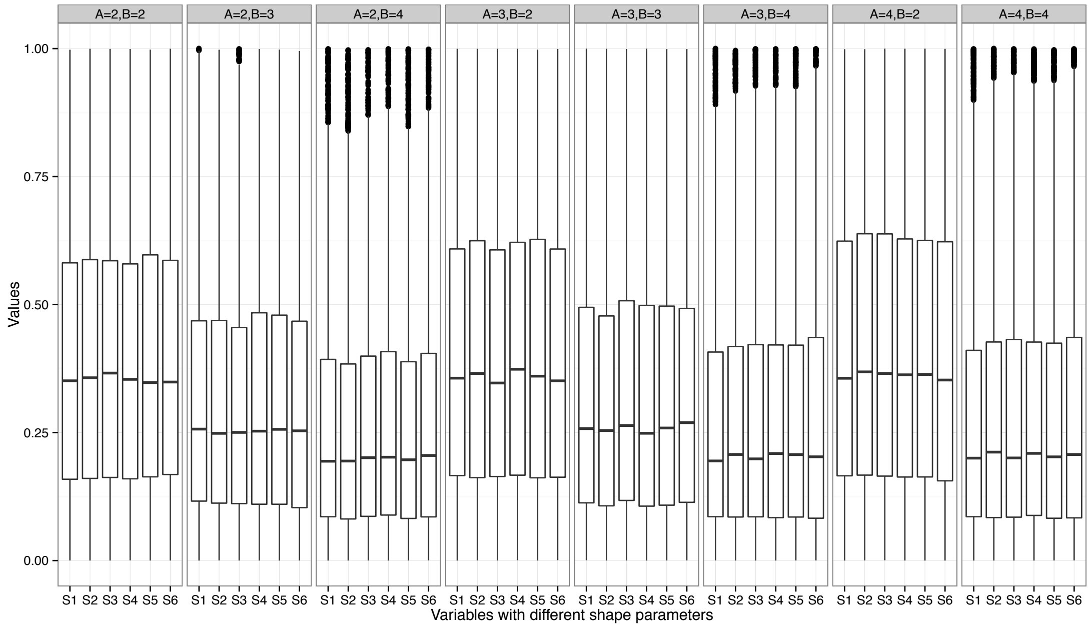

Figure 4: Box-plot showing the distribution of confidence values in 6 artificially generated information sources (S1 - S6) with different shape parameters α (A) and β (B)

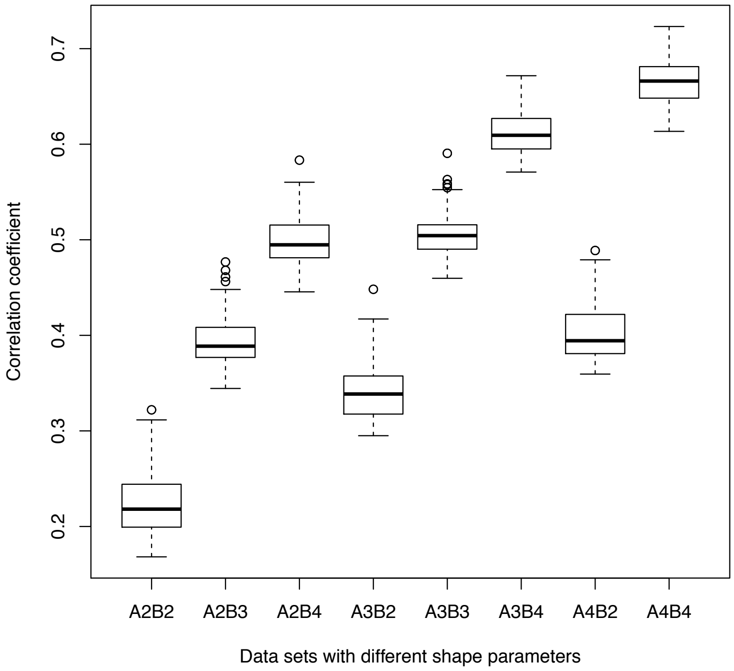

Figure 5: Box-plot showing the distribution of pairwise Spearman rank correlations across 6 artificially generated information sources for different shape parameters α (B) and β (B). Rank correlations were computed for every pair of artificially generated matrices $X^{(i)}$ and $X^{(j)}$

.

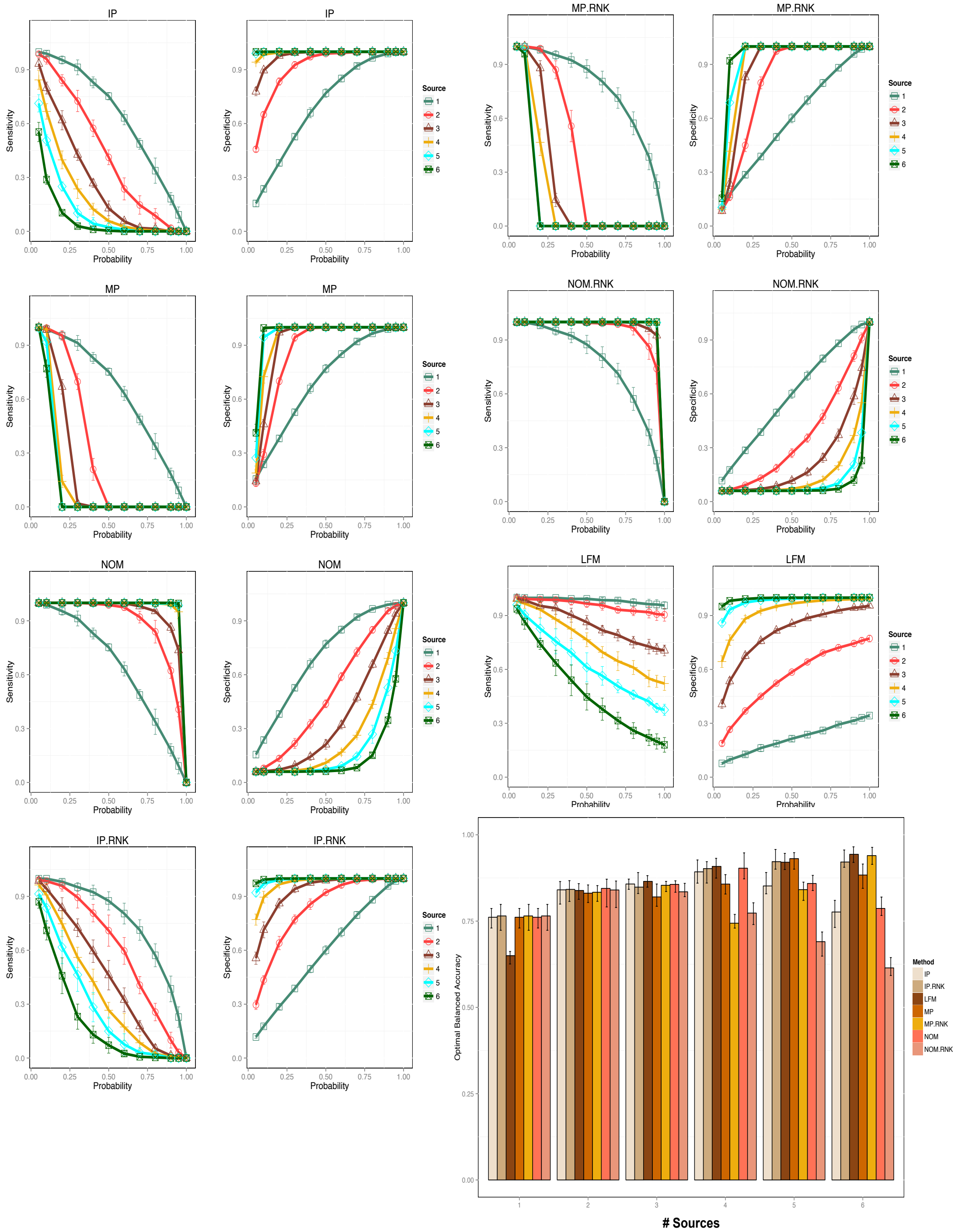

Figure 6: Sensitivity and specificity for different number of artificially generated information sources ($\alpha = 2$, $\beta = 2$). The plot shows the behavior of different priors (top) and the corresponding optimal balanced accuracies (bottom). The number of nodes for all the networks here were 20 (m=20).

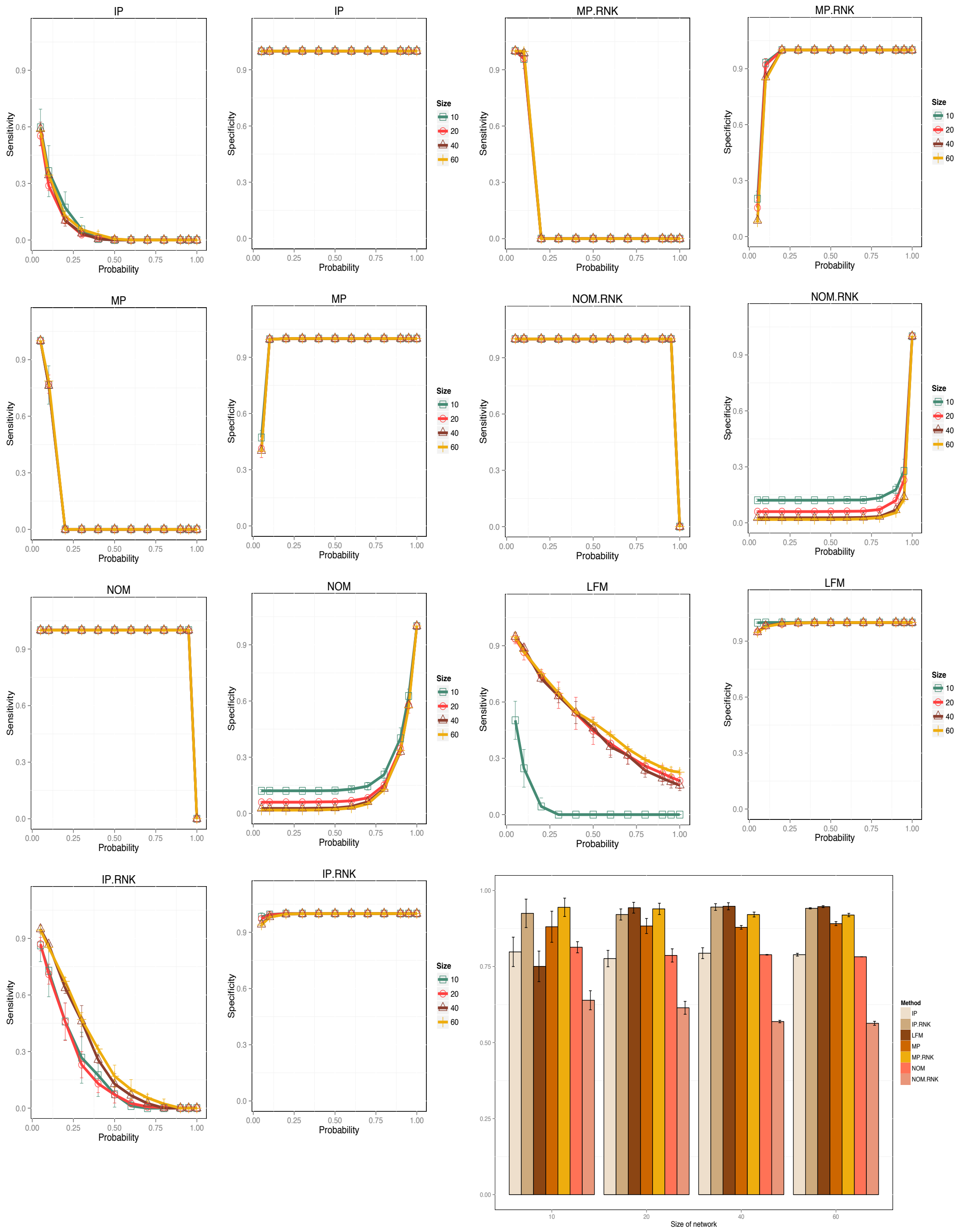

Figure 7: Sensitivity and specificity for different network sizes ($m = 20$, 40 and 60 nodes) for artificially generated information sources ($\alpha = 2, \beta = 2$). The plot shows the behavior of different priors (top) and the corresponding optimal balanced accuracies (bottom). The number of sources for all the networks here were 6.

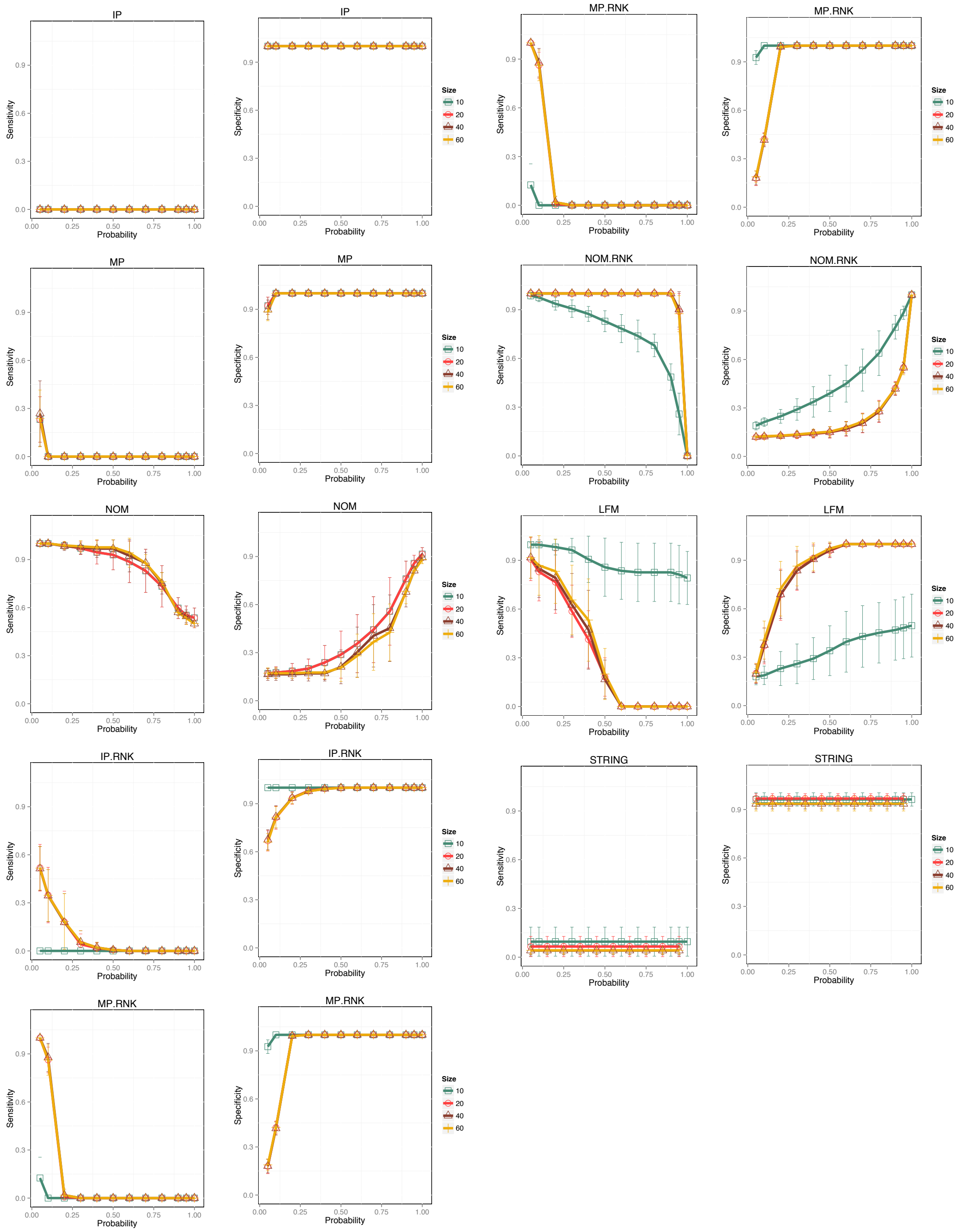

Figure 8: Sensitivity and specificity for different network priors and STRING for KEGG sub-graphs$m = 20$, 40 and 60 nodes (top to bottom). Corresponding oBAC are available in the main document.

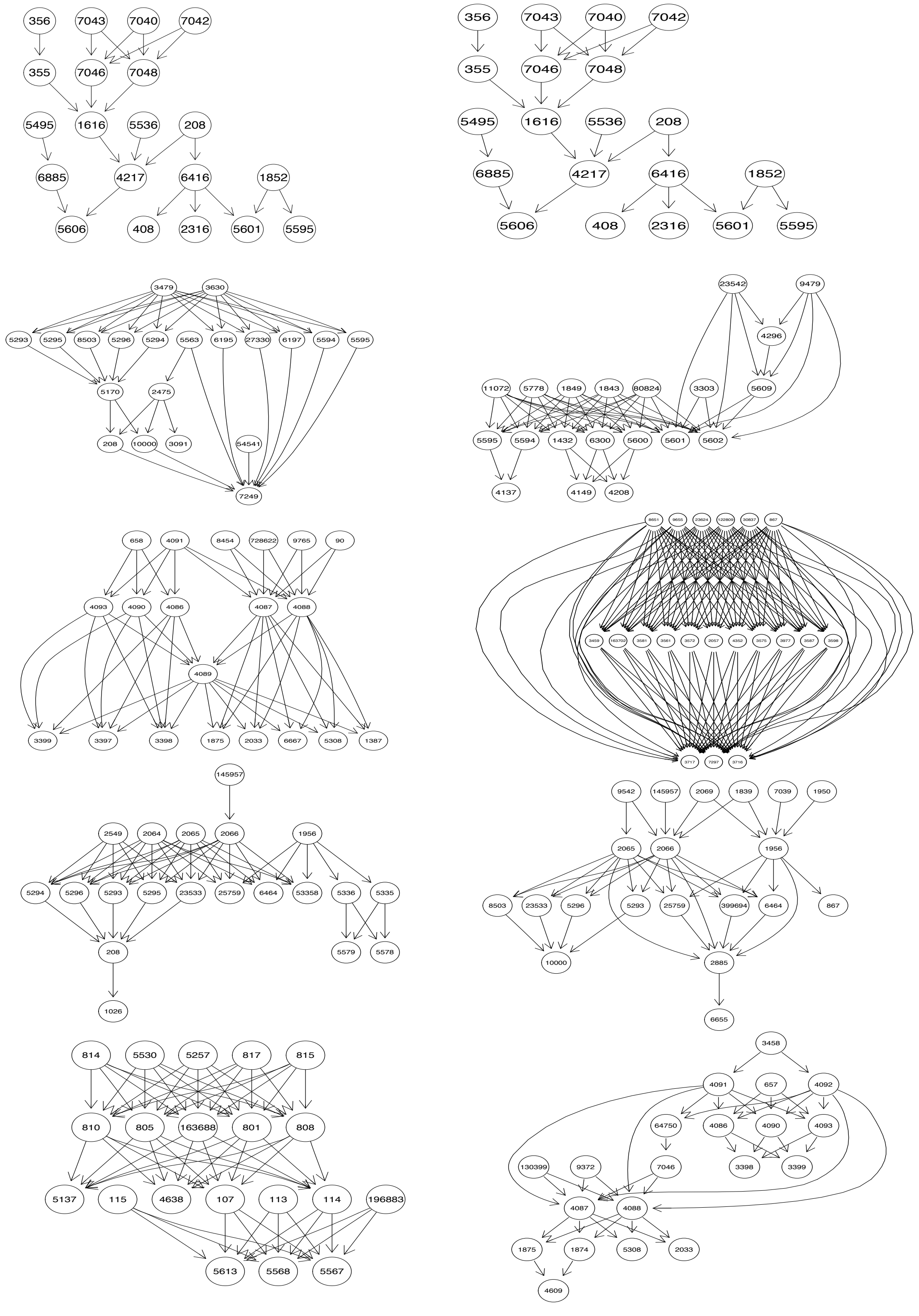

Figure 9: The 10 graphs with # nodes=20 sampled from KEGG via random walks. The node IDs represent Entrez IDs.

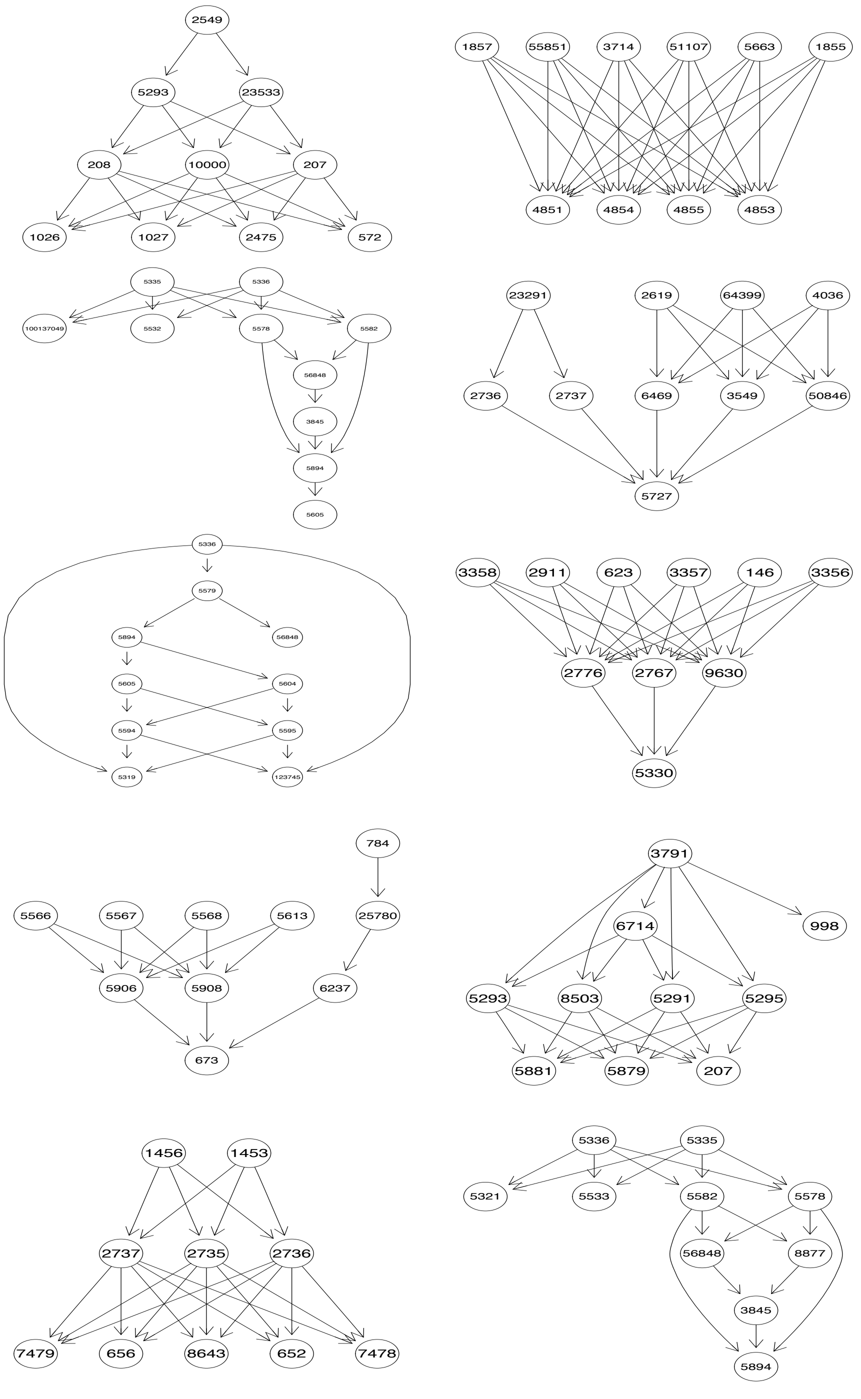

Figure 10: 10 directed acyclic graphs (DAGs) with # nodes=10 selected via random walks on KEGG pathways. The node IDs represent Entrez IDs.

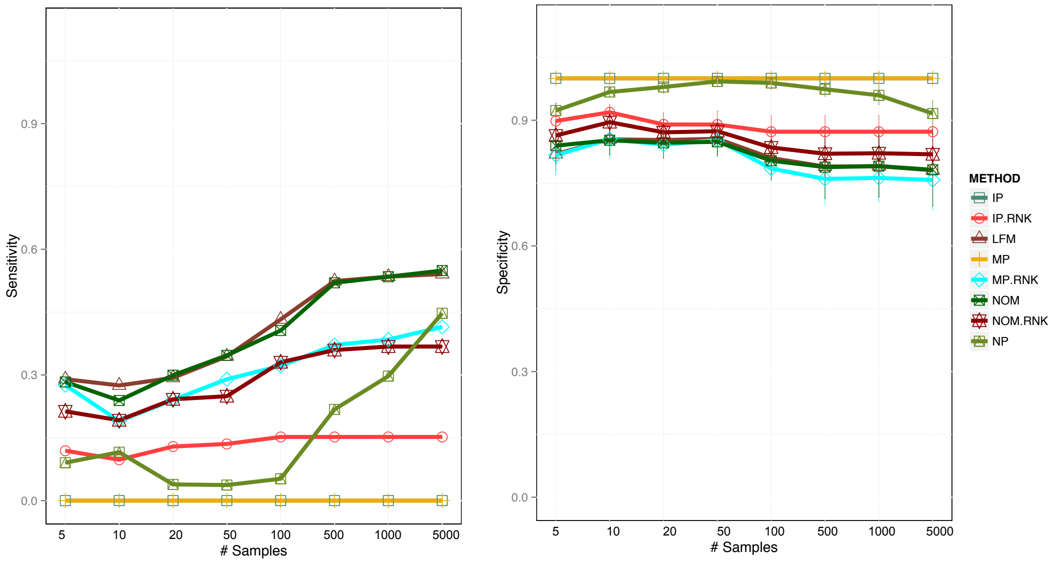

Figure 11: Sensitivity and specificity of Bayesian Network reconstruction for simulated data for a network of 10 nodes ($m = 10$) with different kinds of prior. The corresponding balanced accuracies are shown in the main document

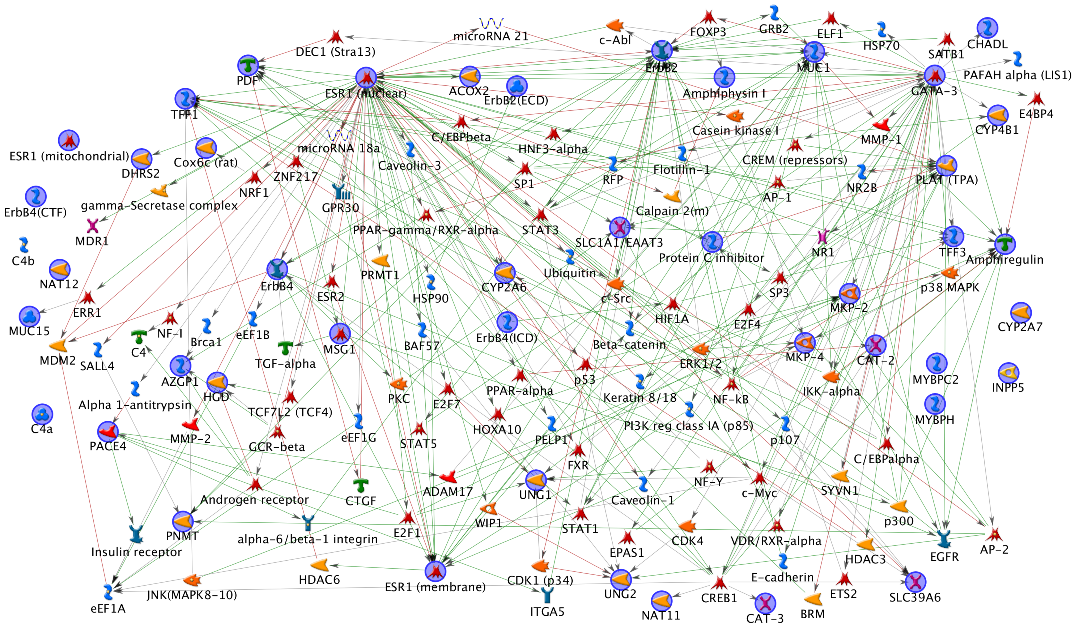

Figure 12: Metacore^{TM}network for 37 selected genes from vant't Veer breast cancer data set. The network was retrieved using the shortest path algorithm (path length of 2). The entire set of interaction with literature evidences are in 'Supplement - 2'

References

[1] Ethan G Cerami, Benjamin E Gross, Emek Demir, Igor Rodchenkov, Ãzagin Babur, Nadia Anwar, Nikolaus Schultz, Gary D Bader, and Chris Sander. Pathway commons, a web resource for biological pathway data. *Nucleic Acids Research*, 39:D685–D690, 2011.

[2] Holger Fröhlich, Mark Fellman, Holger Sültman, and Tim Beißbarth. Predicting pathway membership via domain sinatures. *Bioinformatics*, 24:2137–2142, 2008.

[3] Holger Fröhlich, Mark Fellman, Holger Sültman, Annemarie Poustka, and Tim Beißbarth. Large scale statistical inference of singnaling pathways from rnai and microarray data. *BMC Bioinformatics*, 8(386), October 2007.

[4] Florian Hahne, Alexander Mehrle, Dorit Arlt, Annemarie Poustka, Stefan Wiemann, and Tim Beißbarth. Extending pathways based on gene lists using interpro domain signatures. *BMC Bioinformatics*, 9(1):3, 2008.

[5] Marc Johannes, Holger Fröhlich, Holger Sültmann, and Tim Beißbarth. pathclass: an r-package for integration of pathway knowledge into support vector machines for biomarker discovery. *Bioinformatics*, 27(10):1442–1443, 2011.

[6] R I Kondor and J Lafferty. Diffusion kernels on graphs and other discrete structures. In *Proceedings of the ICML*, 2002.

[7] Dekang Lin. An information-theoretic definition of similarity. In *In Proceedings of the 15th International Conference on Machine Learning*, pages 296–304. Morgan Kaufmann, 1998.

[8] Nicola J Mulder, Rolf Apweiler, Terri K Attwood, Amos Bairoch, Alex Bateman, David Binns, Margaret Biswas, Paul Bradley, Peer Bork, Phillip Bucher, Richard Copley, Emmanuel Courcelle, Richard Durbin, Laurent Falquet, Wolfgang Fleischmann, Jerome Gouzy, Sam Griffith-Jones, Daniel Haft, Henning Hermjakob, Nicolas Hulo, Daniel Kahn, Alexander Kanapin, Maria Krestyaninova, Rodrigo Lopez, Ivica Letunic, Sandra Orchard, Marco Pagni, David Peyruc, Chris P Ponting, Florence Servant, Christian J A Sigrist, and InterPro Consortium. Interpro: an integrated documentation resource for protein families, domains and functional sites. *Brief Bioinform*, 3(3):225–235, Sep 2002.

[9] B. Raghavachari, A. Tasneem, T. M. Przytycka, and R. Jothi. Domine: a database of protein domain interactions. *Nucleic Acids Res*, 36(Database issue):D656–61, 2008.

[10] Andreas Schlicker, Francisco S Domingues, Jörg Rahnenführer, and Thomas Lengauer. A new measure for functional similarity of gene products based on gene ontology. *BMC Bioinformatics*, 7:302, 2006.

Table 1: Pairwise Wilcoxon test for model performance comparison (false discovery rates) for KEGG sub-graphs with $m = 10$ nodes

Methods	IP	IP.RNK	LFM	MP	MP.RNK	NOM	NOM.RNK
IP.RNK	-	-	-	-	-	-	-
LFM	0.0041	0.0041	-	-	-	-	-
MP	0.0203	0.0203	0.0352	-	-	-	-
MP.RNK	0.0423	0.0423	0.0203	0.0304	-	-	-
NOM	0.0041	0.0041	0.2974	0.0041	0.0041	-	-
NOM.RNK	0.0041	0.0041	1.0000	0.0099	0.0041	0.0041	-
STRING	0.0070	0.0070	0.0041	0.0945	0.5111	0.0041	0.0041

Table 2: Pairwise Wilcoxon test for model performance comparison (false discovery rates) for KEGG sub-graphs with $m = 20$ nodes

Methods	IP	IP.RNK	LFM	MP	MP.RNK	NOM	NOM.RNK
IP.RNK	0.0036	-	-	-	-	-	-
LFM	0.0036	0.2712	-	-	-	-	-
MP 0.0144	0.0064	0.0260	-	-	-	-	
MP.RNK	0.0036	0.6250	0.4200	0.0348	-	-	-
NOM	0.0036	0.0036	0.5773	0.0036	0.0091	-	-
NOM.RNK	0.0036	0.0036	0.4648	0.0036	0.0064	0.1022	-
STRING	0.0036	0.0036	0.0036	0.0260	0.0036	0.0036	0.0036

Table 3: Pairwise Wilcoxon test for model performance comparison (false discovery rates) for KEGG sub-graphs with $m = 40$ nodes

Methods	IP	IP.RNK	LFM	MP	MP.RNK	NOM	NOM.RNK
IP.RNK	0.0068	-	-	-	-	-	-
LFM	0.0039	0.0980	-	-	-	-	-
MP	0.0594	0.2166	0.0091	-	-	-	-
MP.RNK	0.0039	0.4038	0.0594	0.0201	-	-	-
NOM	0.0039	0.0495	0.6481	0.0039	0.0039	-	-
NOM.RNK	0.0039	0.0039	0.9219	0.0039	0.0039	0.0091	-
STRING	0.0039	0.0068	0.0039	0.0383	0.0039	0.0039	0.0039

Table 4: Area Under Curve (Standard deviations in brackets) values for KEGG pathway reconstruction with different number of nodes (in brackets are the standard deviations)

Method	10	20	40	60
IP	0.500 (0.00)	0.500 (0.00)	0.500 (0.00)	0.500 (0.00)
IP.RNK	0.500 (0.00)	0.741 (0.03)	0.771 (0.04)	0.779 (0.04)
MP	0.828 (0.04)	0.795 (0.03)	0.803 (0.05)	0.781 (0.05)
MP.RNK	0.833 (0.09)	0.807 (0.08)	0.855 (0.10)	0.882 (0.11)
NOM	0.879 (0.05)	0.875 (0.04)	0.831 (0.04)	0.887 (0.06)
NOM.RNK	0.801 (0.02)	0.881 (0.02)	0.864 (0.01)	0.856 (0.02)
LFM	0.869 (0.05)	0.907 (0.01)	0.918 (0.02)	0.923 (0.03)

Table 5: Pairwise Wilcoxon test for Bayesian Network reconstruction for different sample size (5 to 5000, see main article for details)

sample size	Methods	IP	IP.RNK	LFM	MP	MP.RNK	NOM	NOM.RNK
	IP.RNK	0.85	-	-	-	-	-	-
	LFM	0.11	0.11	-	-	-	-	-
	MP	1.00	0.85	0.11	-	-	-	-
5	MP.RNK	0.17	0.17	1.00	0.17	-	-	-
	NOM	0.11	0.11	1.00	0.11	0.85	-	-
	NOM.RNK	0.17	0.19	0.83	0.17	0.85	0.37	-
	NP	1.00	1.00	0.11	1.00	0.19	0.13	0.27
	IP.RNK	0.703	-	-	-	-	-	-
	LFM	0.032	0.062	-	-	-	-	-
	MP	1.00	0.703	0.032	-	-	-	-
10	MP.RNK	0.437	0.683	0.032	0.437	-	-	-
	NOM	0.272	0.309	0.683	0.272	0.613	-	-
	NOM.RNK	0.227	0.125	0.418	0.227	0.483	0.957	-
	NP	0.026	0.105	0.259	0.026	0.683	0.959	0.905
	IP.RNK	0.422	-	-	-	-	-	-
	LFM	0.041	0.041	-	-	-	-	-
	MP	1.00	0.422	0.041	-	-	-	-
20	MP.RNK	0.049	0.102	0.060	0.049	-	-	-
	NOM	0.041	0.041	0.906	0.041	0.240	-	-
	NOM.RNK	0.049	0.041	0.466	0.049	0.422	0.304	-
	NP	0.304	0.722	0.041	0.304	0.049	0.041	0.049
	IP.RNK	0.922	-	-	-	-	-	-
	LFM	0.016	0.016	-	-	-	-	-
	MP	1.00	0.922	0.016	-	-	-	-
50	MP.RNK	0.016	0.016	0.154	0.016	-	-	-
	NOM	0.018	0.016	0.864	0.018	0.120	-	-
	NOM.RNK	0.046	0.020	0.197	0.046	0.703	0.059	-
	NP	0.197	0.653	0.016	0.197	0.016	0.016	0.026
	IP.RNK	0.625	-	-	-	-	-	-
	LFM	0.029	0.018	-	-	-	-	-
	MP	1.00	0.625	0.029	-	-	-	-
100	MP.RNK	0.102	0.126	0.029	0.102	-	-	-
	NOM	0.031	0.018	0.240	0.031	0.177	-	-
	NOM.RNK	0.029	0.029	0.177	0.029	0.206	0.625	-
	NP	0.102	0.625	0.018	0.102	0.237	0.102	0.031
	IP.RNK	0.675	-	-	-	-	-	-
	LFM	0.016	0.016	-	-	-	-	-
	MP	1.00	0.675	0.016	-	-	-	-
500	MP.RNK	0.092	0.092	0.016	0.092	-	-	-
	NOM	0.016	0.016	1.000	0.016	0.022	-	-
	NOM.RNK	0.038	0.022	0.063	0.038	0.378	0.049	-
	NP	0.016	0.016	0.197	0.016	0.177	0.197	0.799

Table 6: continuation of table 6

sample size	Methods	IP	IP.RNK	LFM	MP	MP.RNK	NOM	NOM.RNK
1000	IP.RNK	0.625	-	-	-	-	-	-
	LFM	0.022	0.022	-	-	-	-	-
	MP	1.00	0.625	0.022	-	-	-	-
	MP.RNK	0.048	0.048	0.025	0.048	-	-	-
	NOM	0.022	0.022	1.00	0.022	0.025	-	-
	NOM.RNK	0.034	0.025	0.048	0.034	0.533	0.048	-
	NP	0.031	0.022	0.424	0.031	0.034	0.424	0.198
5000	IP.RNK	0.675	-	-	-	-	-	-
	LFM	0.014	0.011	-	-	-	-	-
	MP	1.00	0.675	0.014	-	-	-	-
	MP.RNK	0.037	0.025	0.019	0.037	-	-	-
	NOM	0.014	0.011	1.000	0.014	0.019	-	-
	NOM.RNK	0.031	0.019	0.075	0.031	0.957	0.037	-
	NP	0.014	0.011	0.079	0.014	0.011	0.188	0.011
